# Semantic and syntactic interoperability in online processing of big Earth observation data

**DOI:** 10.1080/17538947.2017.1332112

**Published:** 2017-05-31

**Authors:** Martin Sudmanns, Dirk Tiede, Stefan Lang, Andrea Baraldi

**Affiliations:** ^a^ Department of Geoinformatics – Z_GIS, University of Salzburg, Salzburg, Austria; ^b^ Department of Agricultural and Food Sciences, University of Naples Federico II, Napoli, Italy

**Keywords:** OGC WPS, big Earth data, time series, large-scale processing, model-driven semantic analyses

## Abstract

The challenge of enabling syntactic and semantic interoperability for comprehensive and reproducible online processing of big Earth observation (EO) data is still unsolved. Supporting both types of interoperability is one of the requirements to efficiently extract valuable information from the large amount of available multi-temporal gridded data sets. The proposed system wraps world models, (semantic interoperability) into OGC Web Processing Services (syntactic interoperability) for semantic online analyses. World models describe spatio-temporal entities and their relationships in a formal way. The proposed system serves as enabler for (1) technical interoperability using a standardised interface to be used by all types of clients and (2) allowing experts from different domains to develop complex analyses together as collaborative effort. Users are connecting the world models online to the data, which are maintained in a centralised storage as 3D spatio-temporal data cubes. It allows also non-experts to extract valuable information from EO data because data management, low-level interactions or specific software issues can be ignored. We discuss the concept of the proposed system, provide a technical implementation example and describe three use cases for extracting changes from EO images and demonstrate the usability also for non-EO, gridded, multi-temporal data sets (CORINE land cover).

## 1. Introduction

### 1.1. New challenges for big EO data processing

Technical progress in Earth observation (EO) with increasing spatial resolutions of sensors and shorter revisiting times entails the big data slogan to ‘bring the user to the data and not the data to the user’ to the EO community. The quantity and quality of EO data acquired by the new generation of sensors, including the Copernicus Sentinel family, is unprecedented in the history of EO. It is estimated that until mid of 2018 the Sentinel satellite fleet will have collected already more data than the NASA’s Landsat and MODIS fleet together since their launch in the early 1970s (Soille et al. 2016). Although EO data processing has always challenged the technical capabilities of computer processing, existing and future EO volumes confronts the EO community with new challenges, such as establishing workflows for online processing in a cloud-based environment.

Delivering satellite-derived information on mobile devices is crucial to consider them useful for existing and emerging EO data application domains. Examples are agricultural monitoring, disaster management or wildfire detection. Still, despite increasing technical capabilities, downloading and processing gigabytes or terabytes of EO data on thin clients such as a mobile device is cumbersome or not possible at all. Even on thick clients, large-scale multi-temporal analyses are tricky to perform. The volume of input data for analyses may encompass terabytes or even petabytes, data from multiple sensors or data that are acquired under different conditions (Pesaresi 2014). Due to the data volume, variety, velocity and veracity as some of the key aspects of ‘big Earth data’, a more efficient approach for processing EO data is sought in web-based solutions.

To accomplish this, the Group on Earth Observation (GEO) has set the ambitious target to transform EO big sensory data into operational, timely and comprehensive information products (QA4EO task team 2010). Another challenge of GEO, to come up with a functioning ‘system of systems’ is to ‘to allow the access to the right information, in the right format, at the right time, to the right people, to make the right decisions’ (QA4EO task team 2010). The present paper focuses on this second challenge, to bring the information to those who need it – independently from their location, their soft- and hardware and their technical background. This entails a technical challenge as a web-based system allowing a declarative approach to processing big Earth data in full support of both syntactic and semantic interoperability.

In a web-based system, syntactic interoperability means the standardisation of the communication between a software client and a server (Schaeffer et al. 2012). Yet, challenges arising when the system is scaled up. It is not possible as yet to simply plug in a computer to external processing capabilities, as it would be possible to plug it in to the power grid. To cope with large amounts of data every day, a powerful EO data processing workflow makes use of a client-server infrastructure with ‘ready-to-analyse’ data and large computational resources on the server side. The advantage is that clients only send commands to the external processing unit and receive the results. Initiatives in this direction are, for example, the GENESI-DEC project (Cossu et al. 2010) or the ESA G-POD generic grid processing system (Fusco, Cossu, and Retscher 2007).

Semantic interoperability, as defined by the Research Data Alliance (2015) is ‘the ability of services and systems to exchange data in a meaningful/useful way’. It is a more challenging concept because it implies a mutual understanding of the meaning of data and information in the communication process (Harvey et al. 1999). To facilitate semantic interoperability, software clients, which request processing, and servers, which conduct the processing, agree mutually upon clear and distinct understanding of the context and the concepts of entities and their relationships. Another application where ambiguity is unwanted is the sharing workflows within software clients. In the proposed system, this is achieved by means of unambiguous definitions in a world model, which allows software clients, and subsequently human operators, to express interpretation and interpret the results. Many potential users of EO-derived information are non-technical experts and have to rely on processing workflows that were developed by others. These include, for example, farmers interested in the phenology of their cultivations or city planners monitoring the dynamics of urban areas. Here we require domain-specific ‘world models’, that is, domain ontologies, as enabler for semantic interoperability. They are (1) designed as re-usable component of the system and (2) stored and shared in a central storage unit, a so-called knowledge base. The usability of the knowledge base depends on a clear, unambiguous description of its content. Both humans and machines need to ‘understand’ what the world model entails. Semantic interoperability allows different domain-specific world models to be concatenated to a larger workflow.

### 1.2. System design requirements

The design and development phase of the proposed system encompasses two broad aspects, (1), ensuring syntactic and semantic interoperability, and (2) providing an easy-to-use interface for unexperienced users while at the same time supporting more complex tasks from expert users. This translates into five principle requirements for the system to be:

1. agnostic to the client software and client operating system;
2. agnostic or automatically adjustable to the type and characteristics of the client device (desktop, smartphone, etc.);
3. based largely on already existing and standardised technology;
4. able to process any data directly on the database level to deliver the information in near real-time, without having to consider low-level implementation details and having to stick to predefined workflows;
5. supportive and guide standard users and non-technical experts.

Based on these requirements, we propose a scalable, web-based system making use of an OGC (Open Geospatial Consortium) conform Web Processing Service (WPS) for processing EO data in a client-server environment.

**Figure 1.**
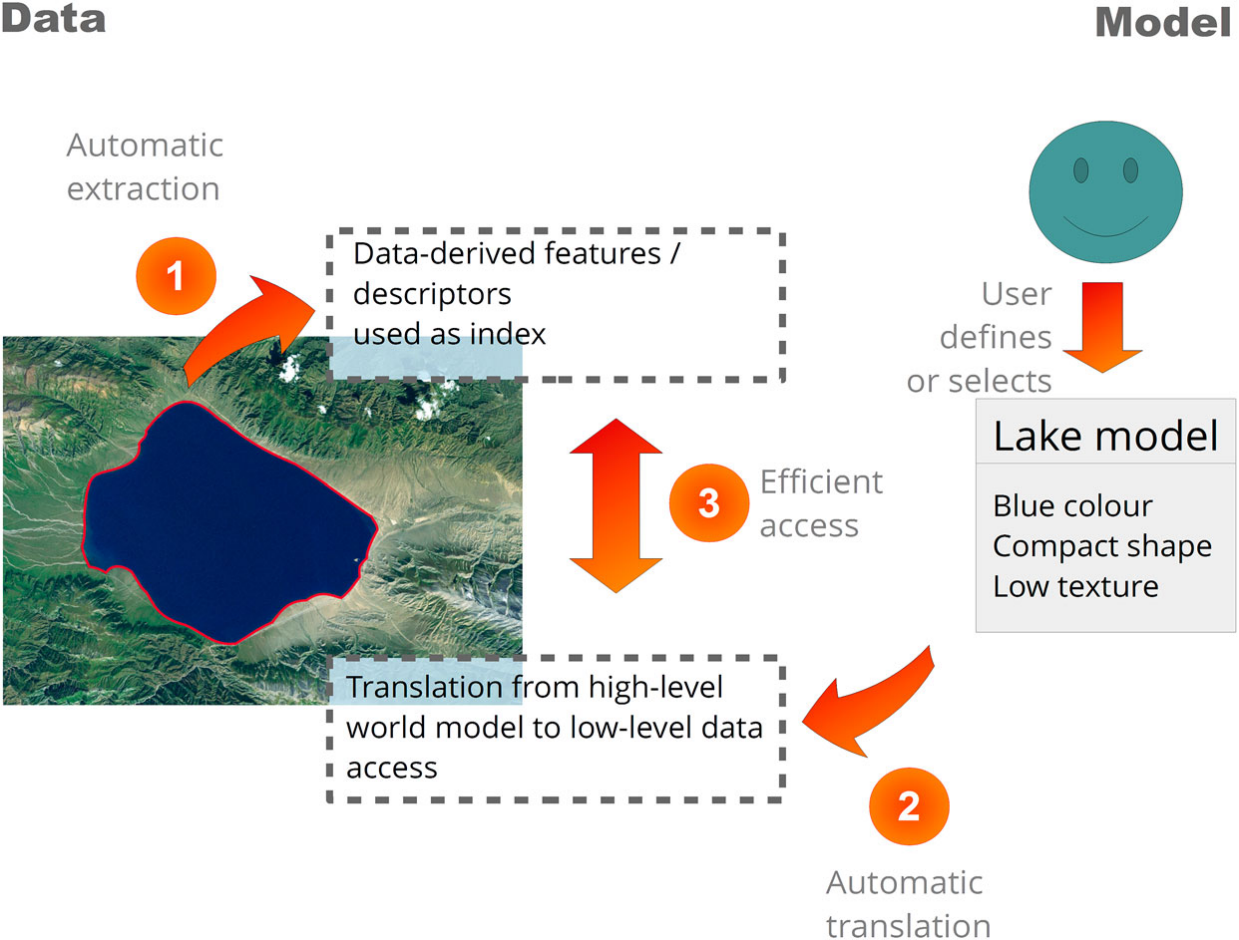
Conceptual view of a model-driven analysis. General features are extracted and stored in a database as index of the EO data (1). A user-defined high-level model of a lake contextualises the features in the information layer. The system decodes and translates the high-level model into a low-level query (2) and executes it against the database (3).

The system is composed of three main components. (1) *Information extraction*. This first component automatically derives information from EO data based on principles of human vision (Marr 1976). The extracted information has a higher semantic level than the original data and is stored in cross-domain information layers. These layers serve as dense index of the EO data content, allowing semantic content-based querying (Tiede et al. 2016). Semantic querying in our system aims to allow users to express their analyses based on their real-world knowledge and in ‘user-speak’. For EO imagery, automatically generated information layers such as a scene classification map, a surface water mask or a vegetation mask can be used. The information layer are generated by a fully automated pixel-based spectral rule-based decision-tree classifier, whose output is a discrete and finite set of semi-symbolic spectral categories, for example, vegetation (Baraldi et al. 2010). The degree of semantic information of these categories is always equal or inferior to that of traditional land cover classes (concepts), which are usually 4D-based (three spatial dimensions and one temporal dimension), for example, deciduous forest. Currently, the proposed system uses a software implementation of this classifier, which is called SIAM^TM^. The integration of these information layers into the proposed systems allows the extraction of additional valuable information from the EO data, for example, based on time-related queries. (2) *Knowledge base*/*world model*(s). The second component allows users to process EO data online by means of expert-based domain-specific world models, which contextualise the information layers. A world model encodes semantic descriptions of real-world structures and characteristics together with their interrelationships based on human reasoning into machine-readable knowledge representations. The model-driven approach has already been introduced in the late 1970s to interpret low-level visual information and changes in images (Marr 1976; Matsuyama and Hwang 1990, 3; O’Rourke and Badler 1980). In this approach, a semantic net describes the entities and their relationships of the objects-of-discourse. (3) *Fact base*. The third component is a centralised storage, the fact base, to which the world models in the knowledge base are connected. Currently, this is implemented as multidimensional array-DBMSs (Database Management System) which instantiates spatio-temporal data cubes (Sudmanns 2016). Figure 1 illustrates the interplay of the different components and the interpretation of EO data by means of a world model.

The proposed system is based on a distributed client-server architecture allowing users to perform semantic model-driven and web-based spatial analyses of EO data. Users develop high-level world models and connect them to the centralised EO data storage, which contextualises the extracted features in the information layers. The underlying system architecture for storing, retrieving, querying and analysing large amount of EO data online is able to outperform traditional desktop-based systems in many spatio-temporal analyses (Sudmanns 2016; Tiede et al. 2016).

### 1.3. Comparison of alternative approaches

In the last years, several technologies and standards, which can be used for online processing of EO data, have been developed and made available to the community (Petcu et al. 2010). Two OGC standards are the Web Coverage Processing Service (WCPS) (OGC 2016a) and the Web Processing Service (WPS) (OGC 2016b), while technology implementations are Google Earth Engine (GEE) (Google Earth Engine Team 2015) or the Jupyter notebooks.

The Open GeoSpatial Consortium (OGC) has issued the WCPS in 2008. Together with the Transactional Web Coverage Service standard, it is one of the two extensions for the Web Coverage Service (WCS) standard. WCPS provides an interoperable interface for processing raster data to handle all kinds of multidimensional raster data, called coverages (Baumann 2010). This enables the transition from a data-driven to a service-driven approach. The WCPS was designed as declarative language executed from a client against a server and is similar to the structured query language (SQL) implemented in relational databases (Baumann 2010). A successful implementation of a WCPS is, for example, provided by Rasdaman and used within the EarthServer project (Baumann et al. 2016). It is further being used by the NASA sensor web suite (Cappelaere et al. 2013) and for web-based land cover mapping as shown by Karantzalos, Bliziotis, and Karmas (2015).

The Web Processing Service (WPS) holds XML-based interfaces for publishing and executing online processing of GIS operations in general. The geospatial semantics makes WPS more suitable than similar approaches, for example, the Simple Object Access Protocol. The 2007 launched WPS standard allows service providers to wrap existing GIS operations in a web service in a service-oriented architecture paradigm allowing for a wide range of applications, from simple calculations to complex workflows (Schaeffer et al. 2012).

The GEE is a proprietary, cloud-based computing environment for processing EO data (Google Earth Engine Team 2015). GEE provides the data (e.g. Landsat or Sentinel imagery) together with a set of algorithms. The GEE allows users to set up a script with Python or JavaScript syntax that runs algorithms on selected datasets. As prominent examples of applications based on GEE the Global Forest Watch Service, a web-based platform for visualisation, analysis and monitoring of forest data (Global Forest Watch 2016) and the Global Surface Water Explorer for exploring long-term changes of surface water areas (Pekel et al. 2016) can be named.

The Jupyter Notebook is an open source and web-based computing environment for data exploration and scientific computing based on narratives (Kluyver et al. 2016). It creates documents, called notebooks, containing code, including results, documentations or comments in Markdown syntax, and visualisations. While it has been launched in the Python environment IPython (Pérez and Granger 2007), the Jupyter project is meanwhile agnostic to the programming language and connects to different interpreters, called kernels. Although Jupyter notebooks have not been designed explicitly for processing geospatial data, it gains popularity in data science and related disciplines. It supports collaborative work as well as access to parallel computing facilitating large-scale processing and complex tasks.

Other examples of web-based platforms, which have been explicitly designed for processing and analysing EO data, include the Amazon Cloud AWS (Amazon Web Service) for processing of Landsat-8 data, with a free access to the Application Programming Interface (API) (Amazon 2016). An example for a commercial product is the ArcGIS Server. It optionally connects to the Landsat images, which are stored in the Amazon Cloud (ESRI 2016). The Australian Geoscience Data Cube (AGDC) is using the National Computational Infrastructure (NCI) to provide Landsat images in the petabyte scale together with processing capabilities over the internet (Evans et al. 2015). The Austrian Earth Observation Data Centre for Water Resources Monitoring (EODC), a collaboration between the technical university of Vienna, the Austrian Meteorological Service (ZAMG) and other companies, pursues a similar approach (Wagner et al. 2014).

To conclude, there is a trend toward online processing approaches, but they are partly competing with each other. This may affect standards more than technological implementations. They provide different levels of user interaction and are not standardised or do not fully provide interoperability.

## 2. Web processing service (WPS) in an integrated EO data online processing environment

### 2.1. Bridging expert users and non-expert users

According to Pinto and Loekken (2016), three levels of interaction with an EO data online processing system exists. At the first level, users apply existing and pre-arranged workflows to a user-uploaded dataset or to a user-defined area of interest (AOI) of an existing dataset. At the second level, the system allows users to arrange different existing algorithms as a new workflow and apply it to a selected dataset. The third and most sophisticated level is the development and subsequent execution of own algorithms. From a user’s perspective, the possibilities of EO data processing are increasing from the first to the third level. However, the more options are available, the more knowledge is required to use the system, including programming skills, when a query language is exposed to the user. We consider the three levels not as mutually exclusive but as coexisting interaction types for a variety of users with different experience.

Our approach establishes permeability of the three levels by using (1) world models, which are collected and shared in a knowledge base, and (2) a collaborative web-based environment as technical basis. It allows users with diverse experiences to extract and interpret information from EO data by having access to expert or crowd sourcing-based generated and shared knowledge. For example, non-expert users are able to perform semantic analyses of EO data using workflows that have been generated by others, similar to the concept of eScience where different users are collaboratively working together in a web-based environment (Hey and Trefethen 2002; Petitdidier et al. 2009). The idea behind eScience is to provide a system, which serves as work sharing and integration platform for multiple users with different competences. The users of these systems may be connected in the physical world as well, for example, when they are working in the same team in the same institute. They may as well be distributed around the world. Consequently, the proposed system should serve as a platform where users can exchange world models as well as workflows and use them to perform online EO data analysis.

Three central properties distinguish the proposed system from traditional desktop-based systems. (1) It reduces redundancy when users share the datasets, their world models and their results. The centralised data storage no longer needs to move large data volumes or distribute the same data to clients. (2) Users share it in a publicly available knowledge base. Therefore, as by-product of solving the actual EO data processing task and its related real-world problem, the system is crowd-sourcing ‘knowledge’. Using the system will incrementally populate the knowledge base and subsequently increase the systems’ internal intelligence. In line with Linus’ law that if enough eyes are looking at a piece of source code, all bugs and errors will be eventually detected (Wang and Carroll 2011), it can be stated that if enough brains with different competences are thinking about problems together, more difficult problems can be solved. (3) It allows users with less experience to extract important information from EO data as they can make use of the collectively gathered world models. Thus, the system serves as connecting element between users and non-expert users to in using EO data. This will make use of the vast amount of valuable information otherwise hidden in EO data archives.

### 2.2. Orchestration and interplay of models, queries, tasks and protocols

An online processing system for EO data is a multi-user system where the conception and maintenance of a highly consistent and meaningful knowledge base is crucial for its success. Users who create world models may not be the ones who are using it later on. Therefore, special attention is paid on the integrity of the knowledge base and the understanding of other users’ world models. The idea is that a user creates a world model in the web application and stores it in the knowledge base. Then, the world model is registered by the system. Another user, either via the provided web application or via any other WPS-compliant client, can use the world model to investigate the database content and extract information. To meet integrity of the knowledge base, the system treats creating and using the world models as separate steps with distinct characteristics.

The proposed procedure differentiates distinct elements with individual specific constraints and is in general oriented at the Jupyter notebook’s prosaic approach. This means that the procedure guides the user through the world model generation and human-readable descriptions of the individual elements as well as the overall documentation makes up a significant part of the querying. The smallest unit is the *world model* itself, which can be generated within the graphical user interface (GUI) of the web application by one or more expert users. During the development stage, several constraints can be laid out to limit its application, for example, to geographical areas, acquisition time and in case of EO images the sensor resolutions, etc. A world model becomes a unique *task* as soon as a user applies it to a spatio-temporal subset of a specific dataset as defined by an AOI as spatial and a duration of interest (DOI) as temporal subset, and executes it against the query engine. Every task runs in a stateless manner and has a unique identifier and status flags associated with it. A client application can use the identifier to check the progress and revisit the result or investigate errors that have occurred.

The development phase contains additional elements. Developing a meaningful and rather complex world model requires testing and revising of numerous versions. Thus, the knowledge base also maintains the individual snapshots that are versioning the world model during its development. This is stored in a world model *protocol*. To provide the lineage of the world models, the connections of each world model to its associated protocol are always maintained. Connected to the protocol of a world model are further metadata including a user-defined name, the time, username and the dataset to which the world model was applied. Users can additionally provide a human-readable documentation for each step. To summarise, a protocol is a set of versioned tasks, which were executed successfully during the development of the world model and its associated metadata.

This procedure guarantees that it is always possible to relate a world model to one or more protocols. It allows insight of how a world model was developed, including its different versions. For scientific work as well as practical applications, maintaining such a protocol is of significant relevance to ensure the quality and the consistency of the knowledge base. Examples when versioning is required are to understand why a world model does not provide correct answers for a selected AOI or to detect mistakes and errors.

### 2.3. Developing and sharing a spatio-temporal world model

The design principle of the web application for creating the world models and their protocols is based on three conditions. (1) It should assist expert users in developing and revising complex world models. (2) Every world model is open source, reproducible and comprehensive, meaning it should be as transparent as possible. (3) The development of a world model is agnostic to the data acquisition system, the data model and data structure of the storage system. The system translates the world model into data specific queries using, for example, the sensor transfer function for EO images (Belgiu et al. 2016). Thus, the same world model allows investigating EO data that are stored across multiple database instantiations and were generated by different acquisition systems.

**Figure 2.**
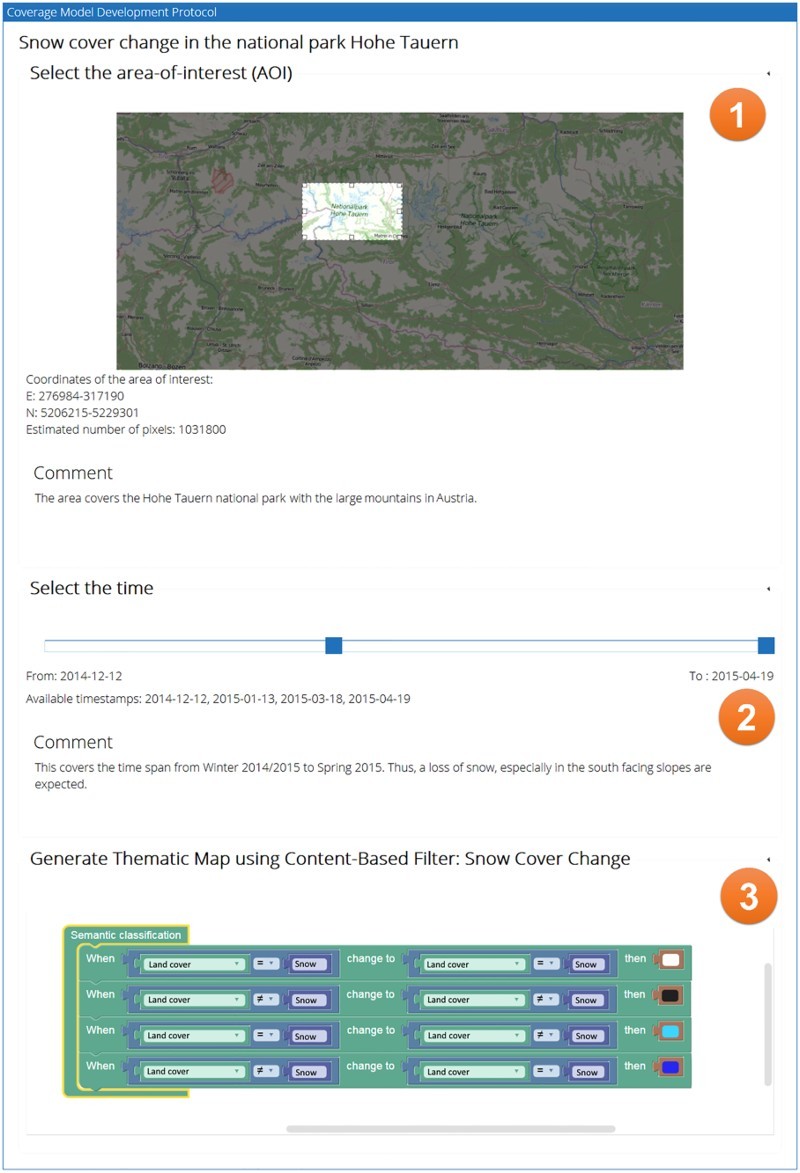
Example of a prosaic query protocol in the web application for creating world models to facilitate semantic online geoprocessing of EO data.

To illustrate the workflow of creating a world model, assume a user might be interested in investigating snow cover changes in the Alps using EO images for which an appropriate world model does not yet exist in the knowledge base. They can choose to either fork an existing and similar world model and continue with it or start from scratch to create a new one. In either case, the system will create a new protocol panel with different visually separated, but internally connected sections (Figure 2). These sections provide interfaces for the selection of the geographical area (1), the time span (2) and the world model (3). In this example, the world model is defined as rules, which can be expressed graphically in the web front end using the Google Blockly JavaScript library. The semantics of changes of the snow cover are in this example defined as different combinations of land cover class changes. Besides the graphical representation, a machine-readable XML representation is generated, which contains the same information. If the newly developed world model is syntactically correct and has been successfully executed at least once on a subset of a dataset, it is possible to share it in the knowledge base, which is a relational database storing the collection of XML representations of world models, including their associated metadata. Therefore, if a world model is shared, it can be found by other users and can be used in WPS execute operations.

### 2.4. Using a world model in a WPS client

**Figure 3.**
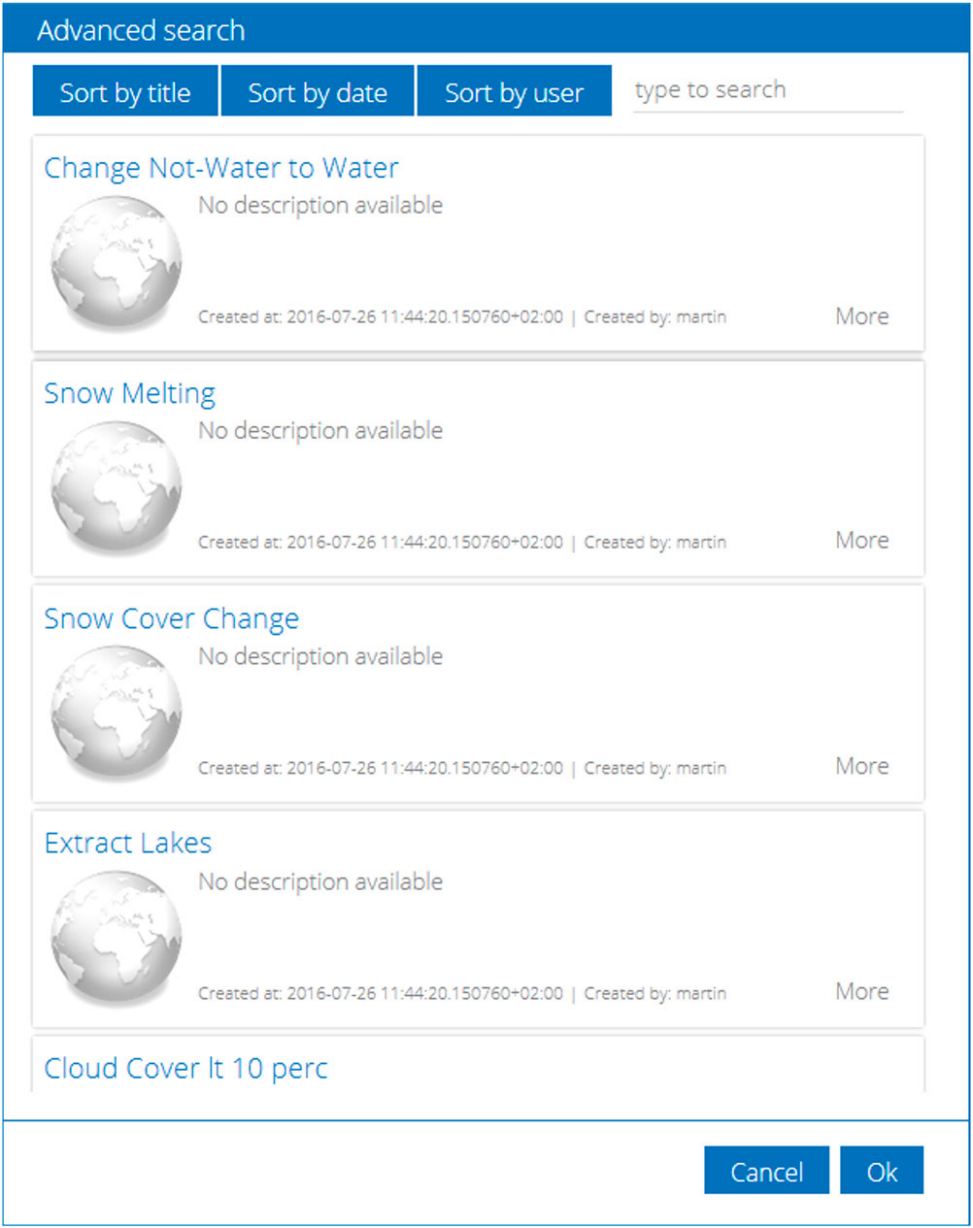
Advanced graphical search in the knowledge base with several sorting and filtering capabilities.

The proposed online processing system provides an API to access the knowledge base and to search for appropriate published world models based on different criteria. The API call returns structured metadata that can be shown as basic information, but also as detailed information for advanced search options. This is especially relevant, when the knowledge base is growing. Figure 3 shows an implemented example interface GUI, structuring the results from the API call. However, access by alternative clients with different capabilities is always possible.

Once the user has selected an appropriate world model, the client receives its XML-based definition. The client configures a WPS operation by setting the world model representation together with additional settings as parameter. The following WPS parameters need to be set: (1) the unique name of the dataset to which the world model is applied, (2) the user-defined AOI (spatial subset) of the dataset, (3) the user-defined DOI (temporal subset) of the dataset and (4) the world model itself.

**Figure 4.**
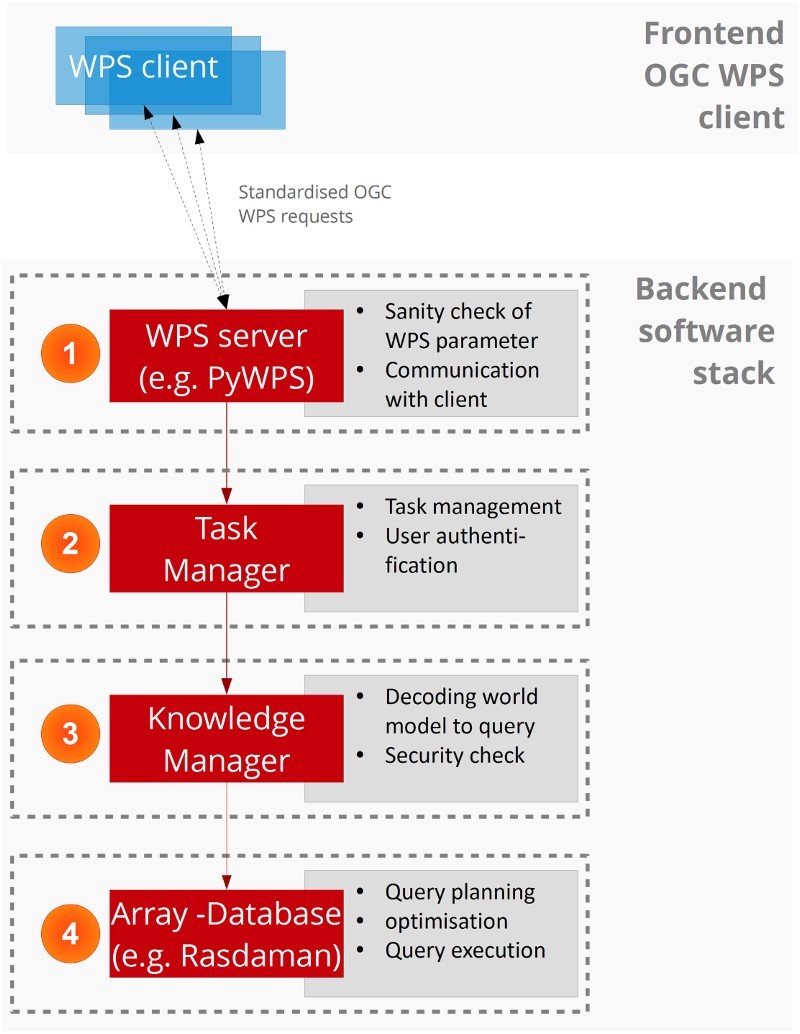
Interplay of components and integration of the OGC WPS as enabler for syntactic interoperability for large-scale online EO image processing.

The defined and configured WPS request is sent to the WPS server, which is the first level of the backend software stack (Figure 4). At first, the WPS process checks whether all WPS parameters are set with reasonable values (1). This is done with the PyWPS server, which is a python implementation of the WPS standard (Čepický and de Sousa 2016). For example, it checks whether the AOI is set and the selected dataset exists in the database. Afterwards, it hands it over to the task manager, which authenticates the user, checks the session validity and instantiates a new task. It then forwards it to the knowledge manager, which connects to the relational database containing the knowledge base and translates the world model into an EO image database query, specifically for the selected dataset (2). It takes the textual XML representation of the world model, divides it into query primitives and composes actual database queries – in this case the Rasdaman query language rasql. After building the query, it performs a set of predefined checks to detect syntax errors of the database query and to identify whether the query could have been manipulated for an intentional or unintentional attack against the server infrastructure. Both, the task manager and the knowledge manager are python programmes, which have been developed in-house. Eventually, the knowledge manager sends the query to the database (here: Rasdaman) and awaits the result, relying on the database engine to perform the actual query (3). Once the query could be evaluated successfully by the database, or an error is reported, the WPS server notifies the WPS client.

During the execution, the task manager populates the metadata database. To increase the user experience, the task is associated with the registered user. Examples for increased user experiences are that users can revisit past queries in their user area of the web application and share query protocols with other users, for example, to receive help from expert users. It stores all metadata including the start time and end time and the number of cells, which have been processed.

### 2.5. System architecture overview

In our approach, the system architecture solves three major problems. It provides a centralised EO data storage system where all users have access. Then, it allows multiple clients to execute processes online and concurrently using standardised OGC WPS services. During the execution, the system translates the high-level world models into low-level queries that consider the characteristics of the data model and sensor system. The system provides a structured interplay of the WPS services and an array-DBMS, which is used as storage backend. This approach provides EO data processing capabilities together with the ‘ready-to-analyse’ EO data and therefore reduces data movement as key enabler for quick response time to queries.

As the system is designed to be able to handle all kind of gridded EO data efficiently and flexible, the data are stored in an array-DBMS. The array-DBMS Rasdaman (Baumann et al. 1998) has been selected for the prototype implementation. Associated metadata, which is required to operate the system, for example, the spatial reference, access rights or task metadata, is stored in an object-relational PostgreSQL database. The array-DBMS storing the information layers instantiates spatio-temporal data cubes with OGC compliant spatial and temporal coordinate reference system definitions.

Spatio-temporal data cubes are an efficient approach to facilitate quick queries through space and time. In a spatio-temporal data cube, the third dimension is time, which is defined in a 1D temporal reference system that overlays the 2D spatial reference system. In contrast to 2D images with its pixels, the atomic elements are voxels. The voxels itself can consist of an array that stores values of the thematic dimension. The data cube model in general has been proven to be scalable and reliable in various operational applications and implementations (Evans et al. 2015; Baumann et al., 2016).

Besides the mentioned data cubes as data model, array-DBMS provide several advantages for the management of large volumes of EO data. Similar to standard relational databases with its SQL, array databases can be queried using declarative query languages. The database approach allows investigation and optimisation of storage-related characteristics, such as indexing, tiling and horizontal scaling as well as data models independently of the application layer. To improve I/O (input/output) performance, the DBMS automatically partitions the data cube into tiles with the same dimensionality. Additionally, array-DBMSs such as Rasdaman (Baumann et al. 1998), SciQL (Kersten et al. 2011) or SciDB (Stonebraker et al. 2013) allow horizontal scaling, that is, distribution of the data cube on multiple server instances to facilitate load balancing and achieve a better performance. For example, such a set-up can evaluate a query in parallel, when it requires accessing data on different servers, and therefore could contribute to a decreased response time.

In order to query the database using storage-independent world models, the latter need to be translated into database queries. The world model can be developed in the web front end by means of a graphical ruleset, which is also represented internally as XML. The XML contains parameter for the positions of the elements for the graphical representations, their meaning (e.g. which classes are selected) and relations (e.g. changes as temporal relation). The in-house developed knowledge manager parses the XML-encoded world model, translates the individual world model components into query snippets and compiles them into a full and valid database query. The query is then executed against the database and for debugging purposes stored in the metadata database.

## 3. Use case examples

The proposed system is able to process any raster data that were inserted into the database. Two use cases using time series of EO imagery and a land cover sequence are presented to illustrate the application exemplarily.

### 3.1. Use case 1: EO image processing

**Figure 5.**
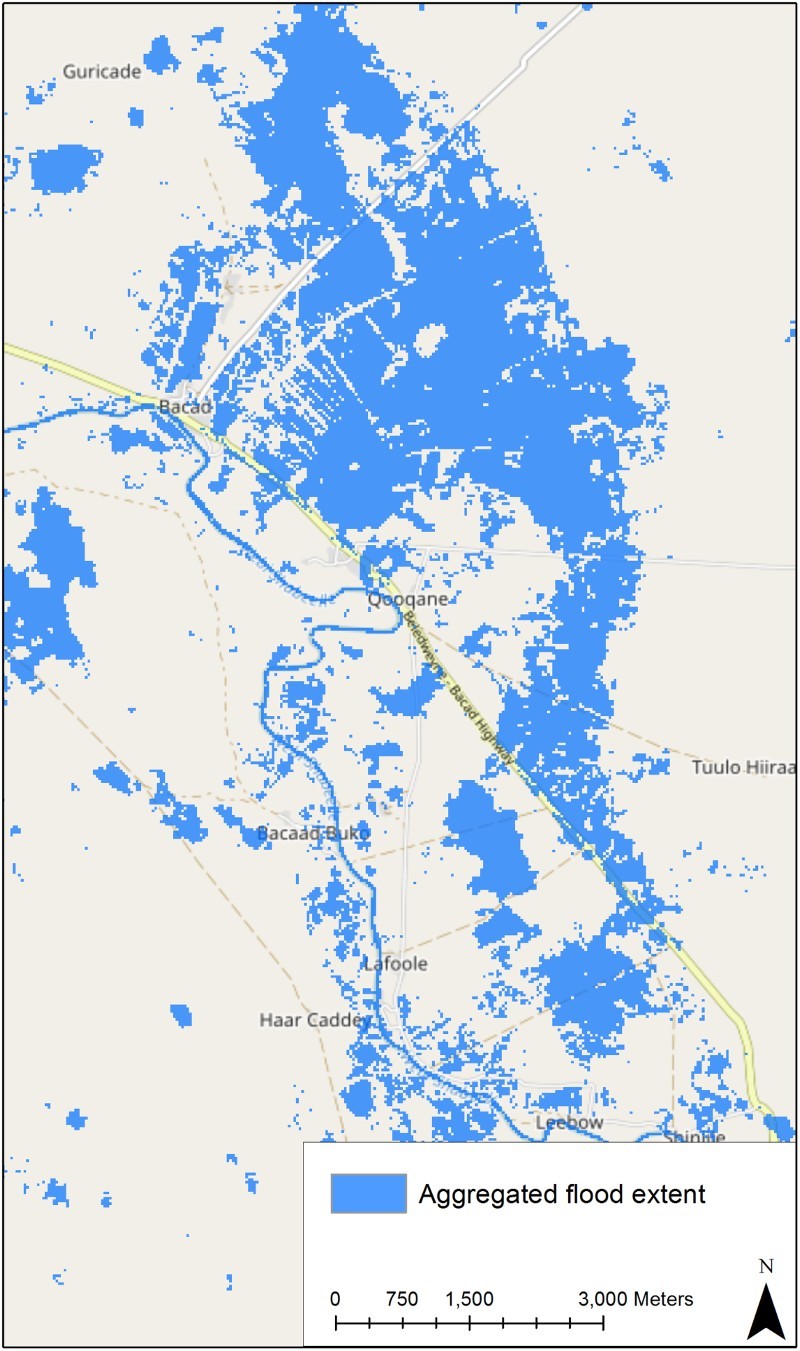
A thematic map showing areas (blue) in the northern part of Somalia, which were flooded at least once in the time between January and September 2016. The information product was generated automatically and online using all available Landsat 8 images of that area in the given time span. The basemap is copyrighted by OpenStreetMap contributors and available from https://www.openstreetmap.org/.

**Figure 6.**
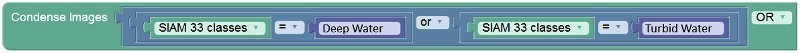
Graphical representation of an example world model that condenses a condition (‘turbid water or deep water’) over time using ‘OR. ‘OR’ generates a binary mask where are all areas where this condition is true at least once are labelled with 1, otherwise with 0.

All available Landsat-8 scenes (78) covering an area in Somalia (path/row: 164/057) in a time span from April 2013 until September 2016 were fully automatically pre-classified using a knowledge-based approach (Baraldi et al. 2010) implemented in SIAM^TM^. Based on the 78 time stamps, a spatial (AOI) and temporal subset (DOI) can be selected and a world model can be applied. Figure 5 shows the result of an example for such an analysis: the maximum water extent for a time period January 2016 to September 2016 is shown comprising areas which were flooded at least once. Flooded areas are calculated by aggregating all pixels through time which showed at least once the spectral signature of water in the given time interval. This is expressed in a world model which graphical representation is shown in Figure 6.

The result is a georeferenced information product according to the world model, ready to be visualised or integrated in additional analyses. Since the world model is stored in the knowledge base, the analysis can be repeated using a different AOI and/or a different DOI.

### 3.2. Use case 2: CORINE land cover (CLC)

The second use case shows the integration of multi-temporal information layers from different non-EO data sources. The raster version of the CLC data sets from 2000, 2006 and 2012 covering the countries Austria, Switzerland and Germany are used. CORINE (Coordination of Information on the Environment) is a European-wide programme initiated in 1985 by the European Commission. The standard CLC nomenclature includes 44 land cover classes (Büttner and Kosztra 2007), grouped in a three-level hierarchy. The level-three subclasses (finest granularity) and some key shape parameters per class were fed into the system.

**Figure 7.**
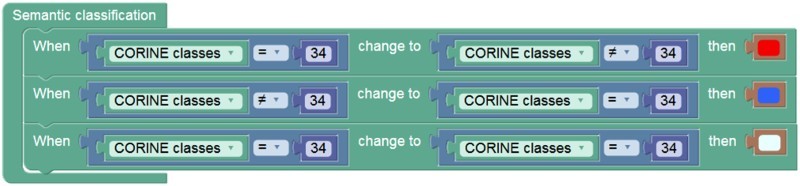
Graphical representation of an example world model that classifies changes in land use classes.

**Figure 8.**
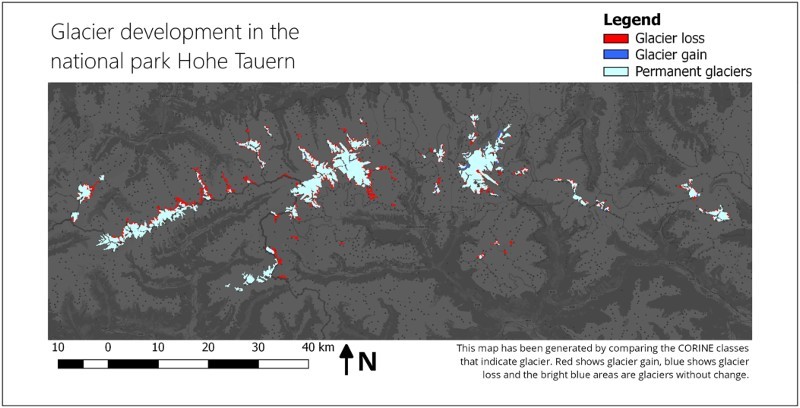
A thematic map showing glacier developments in the Hohe Tauern national park. The information product was generated automatically and online using CLC/land use classes. The basemap is copyrighted by OpenStreetMap contributors and available from https://www.openstreetmap.org/.

The transitions from the CLC class ‘glaciers and perpetual snow’ (CLC class = 34) to any other CLC class (CLC class! = 34) or vice versa queries the development of glaciers over the temporal extent of the CORINE datasets. The graphical representation shown in Figure 7 creates new classes based on the identified changes. Here, the new classes that indicate ‘potential glacier loss’, ‘potential glacier gain’ and ‘persistent glacier’ are encoded with user-defined colours to be used in the thematic map. The information product of the thematic map (Figure 8) generated ‘on-the-fly’ highlights the query result according to the world model being applied and indicates where the respective phenomenon occurs.

**Figure 9.**
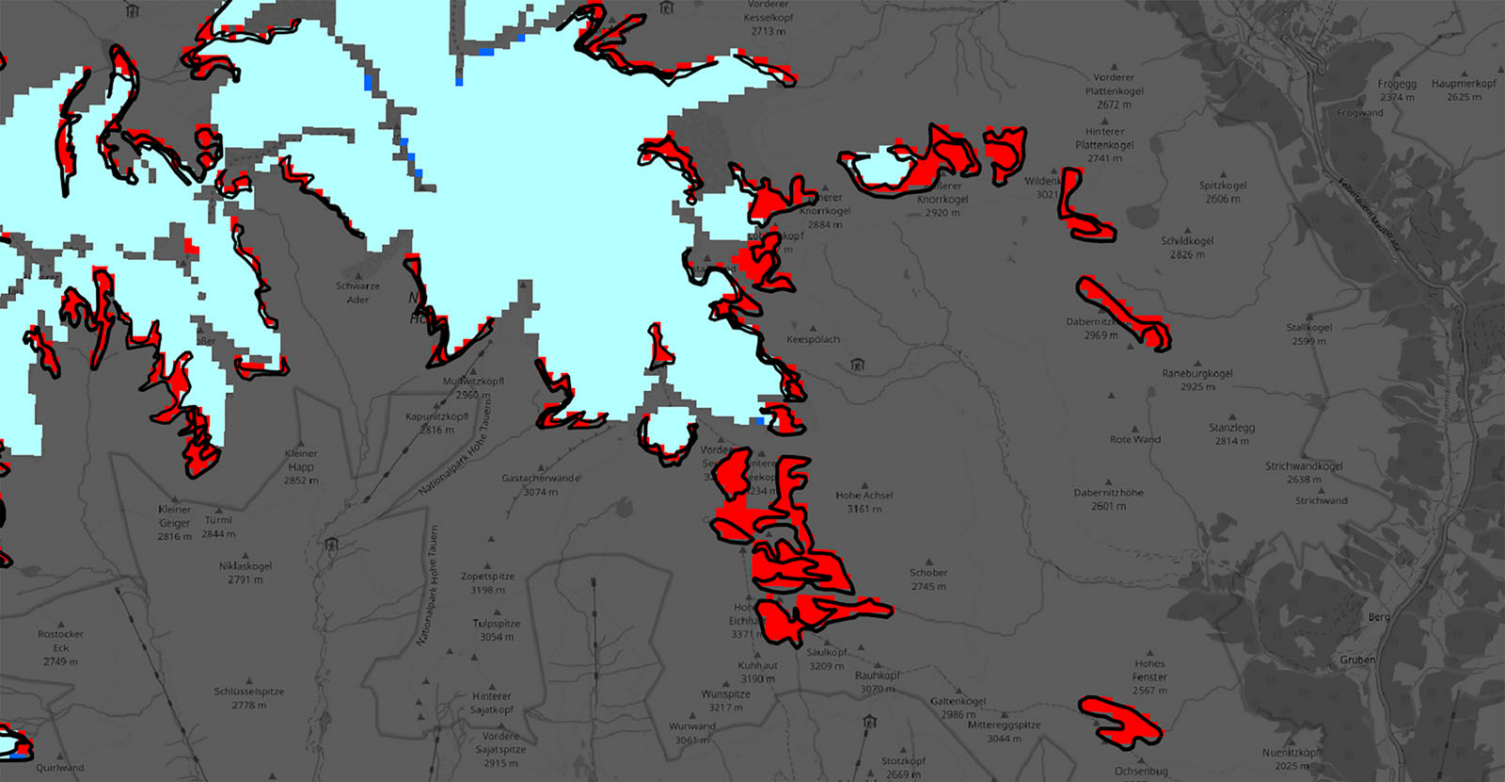
Detail view of a comparison between the extracted results and the official CORINE change layer with black outline. The basemap is copyrighted by OpenStreetMap contributors and available from https://www.openstreetmap.org/.

Figure 9 shows a comparison of a subset between the automatically extracted areas and the official CORINE change layer shown with black outlines. Visual analysis reveals a high match between the objects, while there are some deviations in the outlines specifically.

**Figure 10.**
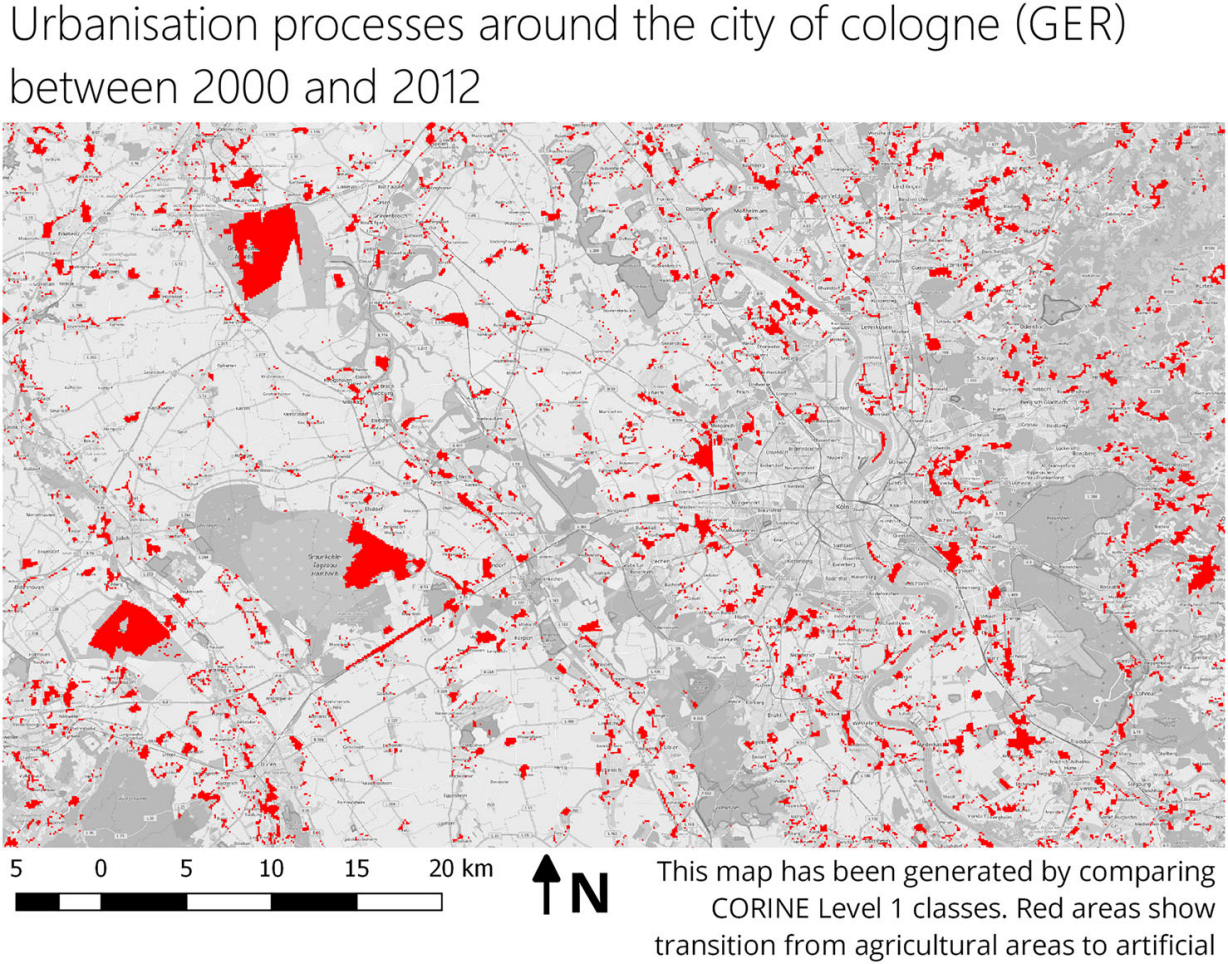
A thematic map showing urbanisation processes around Cologne (Germany). The information product was generated with the same physical dataset than Figure 8 but applying a different world model. The basemap is copyrighted by OpenStreetMap contributors and available from https://www.openstreetmap.org/.

A second example is the extraction of urbanisation processes. Based on the assumption that this can be expressed using the CORINE level 1 classes, all areas that changed from agricultural areas to artificial surfaces can be extracted. The thematic map in Figure 10 shows the result of this query. A possible extension is to include the shape information to extract only large, connected areas.

**Figure 11.**
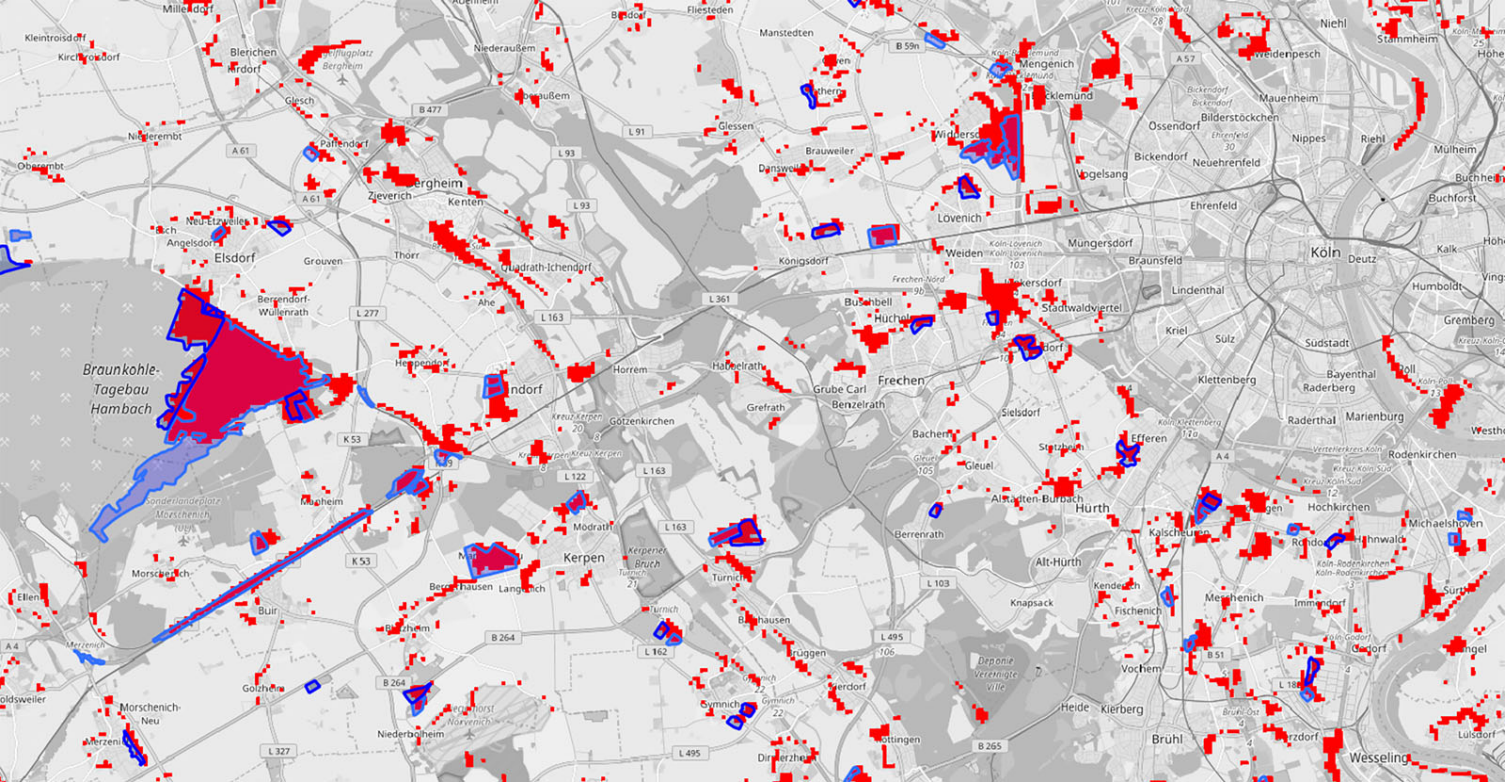
Detail view of a comparison of extracted features from the remote dataset and the official CORINE change layer (blue areas). The basemap is copyrighted by OpenStreetMap contributors and available from https://www.openstreetmap.org/.

Figure 11 shows the difference between the automatically extracted areas and the official change CORINE layer. Although many small areas could be detected, which are not present in the official data, major changes are identical. This includes the areas of brown coal mining in the western part, the newly built train station, which is visible as large, elongated object and the larger newly created settlements around Cologne. A detailed comparison with the CORINE change layer is difficult, since the original datasets might be updated during the generation of the change layer (Tiede 2014).

## 4. Discussion

Backbone of the semantic and syntactic interoperability of the proposed system are the eScience-based web application, which communicates with the array-DBMS using a standardised interface, and the capabilities of understanding of knowledge based on human reasoning as well as the subsequent translation into queries against the array-DBMS.

This paper presents an approach that combines characteristics of the standardised interface of the OGC WPS and the prosaic approach of conducting complex analyses of the Jupyter notebooks. It combines older ideas of semantic image processing using world models and the new web-based technologies that became available recently. The strength of the system is using the standardised interface WPS for syntactic interoperability and allowing users to develop the world model in a comprehensive and reproducible way. It therefore enhances the syntactic interoperability of WPS with semantic interoperability. In contrast to WCPS, our approach embeds both machine- and human-readable knowledge as a world model into the WPS request. A knowledge manager translates the world model into a query, depending on the characteristics of data sources and their location. Although the client need to provide some special capabilities to be able to handle the world models, we argue the required effort of extending them is limited since the WPS execution embeds the full world model as parameter.

Users with different levels of expertise are able to benefit from the globally collected and gathered knowledge. Therefore, the system is designed to be adaptable such that it fits to the user. Moreover, exchanging of world models and embedding them into a client-independent WPS brings EO data-processing capabilities to any device. Hence, it is possible to create a world model comfortably on a desktop workstation and subsequently use it on a mobile device in the field.

The injection and translation of knowledge is a crucial point in the workflow. Presented here is an approach that makes use of world models to which protocols are associated during the development. Although this might be complex, it guarantees interoperability and consistency. It is always possible to roll back to any point in the development process of a world model and investigate it. The knowledge representation itself is in XML and therefore agnostic operating systems and devices. This also allows generating different instantiations of algorithms in different programming or querying languages. Therefore, world models do not need to be changed to consider the specific characteristics of the imaging sensors, when underlying storage engine is exchanged or when they are used within different clients.

The proposed system is able to exploit the advantages of storing EO data in array-DBMSs compared to competing approaches such as storing them as flat files. One drawback is the limitation on the capabilities of the DBMS and the expressiveness of its query language. Still, most array-DBMS such as Rasdaman, SciQL or SciDB allow the definition of user-defined-functions which enhances the query language with custom functionalities (Sudmanns 2016).

Three examples illustrated how straightforward it is to conduct analyses using the web-based application. The innovative facet is that they were processed online using a declarative approach. The re-using of the same dataset eliminated the need of data management prior to the analysis. The analyses – due to the described architecture – can be done on-the-fly, depending on the size of the subset and the time span. For the flood mask extraction in the first use case in the given spatial subset (7 km ×  9 km, pixel size: 30 m) and all available images in the selected time stamps, the calculation time is below 10 seconds using a virtual machine with 4 CPUs and 16 GB RAM. To solve all use cases, it was not necessary to download the data over the internet prior to the analysis. The models were developed within the web application and sent to the server using WPS. The server returned only the result images, which were of the size of several kilobytes.

## 5. Conclusion and outlook

The unprecedented availability of EO data and their increased volume, variety and velocity makes it necessary to re-evaluate current image processing practices as well as to guide future technological developments. This paper illustrated a contribution where different users can work together in a collaborative manner online in a client-server infrastructure to extract information form a potentially large amount of EO data. This type of collaboration requires semantic interoperability, which is achieved by developing world models using well-defined procedures. The comprehensive machine- and human-readable world models steadily extend the knowledge base of the system. Further, the same user, or a different user applies the world models to different kind of data sources to perform semantic EO data processing online on a distributed parallel processing environment.

Since the knowledge base of the system is augmented crowd sourcing-based or expert-based, this approach is relying on the participation of many people who are willing to inject and share their knowledge and algorithms. In this facet, it is similar to already existing infrastructure provider such as GEE. However, due to the architecture of the proposed system that allows adjusting the rule sets on the client side, the knowledge base is open source and can be of true advantage to any user.

The proposed system is able to bring from big Earth data derived information to a broader audience. The proposed system in its current version is able to fulfil the above-introduced functional and non-functional requirements by

1. using OS-independent machine-to-machine communication using XML;
2. using the WPS standard for online processing of EO data and therefore supporting multiple clients;
3. its underlying architecture using array-DBMS facilitating distributed querying capabilities;
4. using a crowd-sourcing-based or expert-based approach and comprehensively generated and managed knowledge base supporting inexperienced users as well as expert users.

Future developments aim at incorporating additional Earth-related data sources. Examples are UAV-based (unmanned aerial vehicle) images or data that have been generated by simulation or assimilation. Another subject is the implementation of different abstract data types such as coverages, time series or trajectories how it is defined in Ferreira, Camara, and Monteiro (2014). The underlying data model will be extended from a purely array-DBMS-based approach to an approach that combines raster and vector data, including inter-object relationships. The aim is to support additional queries such as geometric and topological relationships. Several technical developments of the front end of the web application aim at improving the workflow for unexperienced users. This encompasses improving the expressiveness of the world model, for example, by using a fully implemented semantic net as graphical representation. A browser-based visualisation of multidimensional EO data will be helpful to develop the world models. A separation of the development and testing of world models in a sandbox component from their operational in a production component protect the system and allows scaling up applications.
